# The present and future of immunocytokines for cancer treatment

**DOI:** 10.1007/s00018-022-04514-9

**Published:** 2022-09-06

**Authors:** Dennis Y. Gout, Lotte S. Groen, Marjolein van Egmond

**Affiliations:** 1 Department of Molecular Cell Biology and Immunology, Amsterdam UMC Location Vrije Universiteit Amsterdam, Boelelaan 1108, Amsterdam, The Netherlands; 2 Cancer Biology and Immunology Program, Cancer Center Amsterdam, Amsterdam, The Netherlands; 3 Cancer Immunology Program, Amsterdam Institute for Infection and Immunity, Amsterdam, The Netherlands; 4 LUMICKS, Paalbergweg 3, 1105 AG Amsterdam, The Netherlands; 5 Department of Surgery, Cancer Center Amsterdam, Amsterdam UMC, Vrije Universiteit Amsterdam, De Boelelaan 1117, Amsterdam, The Netherlands

**Keywords:** Monoclonal antibody, Antibody-cytokine conjugate, Immunocytokine, Interleukin-2, Immunoglobulin G

## Abstract

Monoclonal antibody (mAb) therapy has successfully been introduced as treatment of several lymphomas and leukemias. However, solid tumors reduce the efficacy of mAb therapy because of an immune-suppressive tumor micro-environment (TME), which hampers activation of effector immune cells. Pro-inflammatory cytokine therapy may counteract immune suppression in the TME and increase mAb efficacy, but untargeted pro-inflammatory cytokine therapy is limited by severe off-target toxicity and a short half-life of cytokines. Antibody-cytokine fusion proteins, also referred to as immunocytokines, provide a solution to either issue, as the antibody both acts as local delivery platform and increases half-life. The antibody can furthermore bridge local cytotoxic immune cells, like macrophages and natural killer cells with tumor cells, which can be eliminated after effector cells are activated via the cytokine. Currently, a variety of different antibody formats as well as a handful of cytokine payloads are used to generate immunocytokines. However, many potential formats and payloads are still left unexplored. In this review, we describe current antibody formats and cytokine moieties that are used for the development of immunocytokines, and highlight several immunocytokines in (pre-)clinical studies. Furthermore, potential future routes of development are proposed.

## Introduction

Following the first description of monoclonal antibodies (mAbs) in 1975 [1], it took only eleven years to develop the first FDA-approved mAb-based immunotherapy. The drug named Muromonab was used to prevent transplanted kidney rejection by targeting CD3 on T cells [2]. Unfortunately, a plethora of side-effects was associated with this therapy, including but not limited to cytokine release syndrome (CRS), central nervous system complications and thrombosis, which ultimately resulted in voluntary withdrawal from the US market by its manufacturer [3]. Some side-effects have been attributed to the murine nature of the mAb. In some cases, anaphylaxis was induced. Additionally, efficiency of these antibodies was reduced, because injection of murine antibodies led to the development of human-anti-mouse-antibodies (HAMAs). As such, the murine origin of mAbs became a major obstacle to achieve efficient new mAb therapies [4]. To overcome this, chimeric, humanized or fully human mAbs have been developed [5]. In chimeric mAbs, the constant regions are of human origin, while in humanized antibodies, the framework regions of the variable regions are also human.

As a result, several effective mAb therapies have been developed, including anti-CD20 mAb therapies to treat several B-cell malignancies and autoimmune diseases (e.g. Rituximab, Ofatumumab) and checkpoint inhibitors (e.g. Pembrolizumab (αPD1), Ipilimumab (αCTLA-4) [6–9]. Nonetheless, other mAb cancer therapies are not effective as a mono-therapy [7]. A possible solution is the combination of mAb therapy with other cancer therapies, such as co-treatment with radio- or chemotherapy. Traditionally, both therapies were administered separately, but nowadays also radionuclide-antibody-conjugates (RACs) and antibody–drug-conjugates (ADCs) are increasingly developed [10, 11]. Furthermore, antibodies have the ability to engage the immune system. They can activate the complement pathway, but additionally can bridge cytotoxic effector cells with cancer cells, through their Fc tail [12]. Unfortunately, many tumors have an immunosuppressive tumor micro-environment (TME) [13, 14], which dampens effective activation of the immune system. As such, combining mAb therapy with agents that focus on changing the tumor micro-environment (i.e. antibody-cytokine-conjugates) may also represent an attractive way to improve efficacy of antibody therapy, and is the focus of this review.

### The immune-suppressive tumor micro-environment

Solid tumors establish their immunosuppressive TME via a variety of mechanisms. A large part of the immune suppression is mediated through tumor-associated regulatory immune cells such as tumor-associated macrophages (TAMs), tumor-associated neutrophils (TANs), FoxP3 + regulatory T cells (Tregs), and myeloid-derived suppressor cells (MDSCs) [15, 16]. In the TME, tumor-associated regulatory immune cells secrete various immunosuppressive cytokines, such as interleukin 10 (IL-10) and transforming growth factor beta (TGF-β), which inhibit immune cell activation. Additionally, they express immune-suppressive surface molecules such as programmed death ligand 1 (PD-L1) and cytotoxic T-lymphocyte-associated protein 4 (CTLA-4), which also induce inhibitory signaling in immune cells [17, 18].

Cytokines with a pro-inflammatory action may counteract immunosuppression in the TME. Several cytokines have been identified as suitable candidates. For instance, IL-2 is important for activation, differentiation and maintenance of cytotoxic effector cells, such as cytotoxic T cells (CTLs), natural killer (NK) cells and macrophages, but can also directly act on tumor cells by inducing apoptosis [19, 20]. Recombinant IL-2 monotherapies have already been tried in patients with metastatic melanoma or metastatic renal carcinoma in the eighties with mild success [21, 22]. Only some patients benefited due to the toxicity that occurred at higher doses, limiting the efficacy of the therapeutic dose. Treatment of metastatic renal cell carcinoma and melanoma with systemic IL-2 therapy specifically increased the number of tumor-infiltrating lymphocytes (TILs) [23–25].

Additionally, IL-12 is a heterodimeric cytokine, composed out of p35 and p40 subunits, and is involved in communication between innate and adaptive immune responses [26]. IL-12 is primarily secreted by antigen-presenting cells after infection. It stimulates interferon gamma (IFN-γ) production by T and NK cells, and shifts CD4+ T cell differentiation toward a Th1 phenotype [27]. MHC-I presentation as well as NK cell and T cell proliferation is stimulated as well, whereas Tregs are inhibited by IL-12 [26, 28]. Moreover, IL-12 also has anti-angiogenic activities [29].

Tumor necrosis factor alpha (TNF-α) is also a robust regulator of immune cells [30]. It binds to the TNF receptor 2 (TNF-R2), that is mostly expressed by immune cells, and promotes migration of CTLs and NK cells [31, 32]. In addition, TNF-α can also be directly cytotoxic to tumor cells by binding to TNF-R1, which is expressed on most tissues. TNF-α acts on endothelial cells to increase vascular permeability and mediates hemorrhagic necrosis [33], which can eradicate the tumor mass by damaging tumor-associated vasculature. Furthermore, TNF-α is able to stimulate upregulation of cell adhesion molecules such as E-selectin, ICAM-1 and VCAM-1 by endothelial cells [34]. This favors influx of pro-inflammatory leukocytes to the cancer site [35].

Thus, systemic pro-inflammatory cytokine therapy represents an attractive option to activate the immune system, but, is unfortunately notorious for its toxicity, as delivery is untargeted [36, 37]. Hence, by conjugating cytokines to mAbs, efficacy and safety of both therapies might be increased. On the one hand toxicity of cytokine treatment may be lowered, as cytokines are specifically delivered at the cancer site. On the other hand mAb therapy may become more effective as effector immune cells will be simultaneously activated by both cytokine and antibody [38, 39]. Antibody-cytokine-conjugates, otherwise known as armed-antibodies or immunocytokines, were originally developed as a vehicle for the local delivery of lymphotoxin or TNF-α to solid tumors in an attempt to increase their half-life without dramatically increasing toxicity [40, 41]. Over the years, various innovations in both the fields of antibody engineering and cytokine therapy have allowed the development of immunocytokines with a plethora of antibody formats and an increased number of their potential armaments. This wide variety of antibody formats and payload moieties allows for great flexibility in designing an immunocytokine to treat a specific disease or to overcome a specific problem.

In this review we provide an overview of the antibody formats and cytokine moieties that are currently used for the development of immunocytokines. Furthermore, potential future routes of innovation are addressed.

## Antibody formats

### Intact IgG

**Fig. 1:**
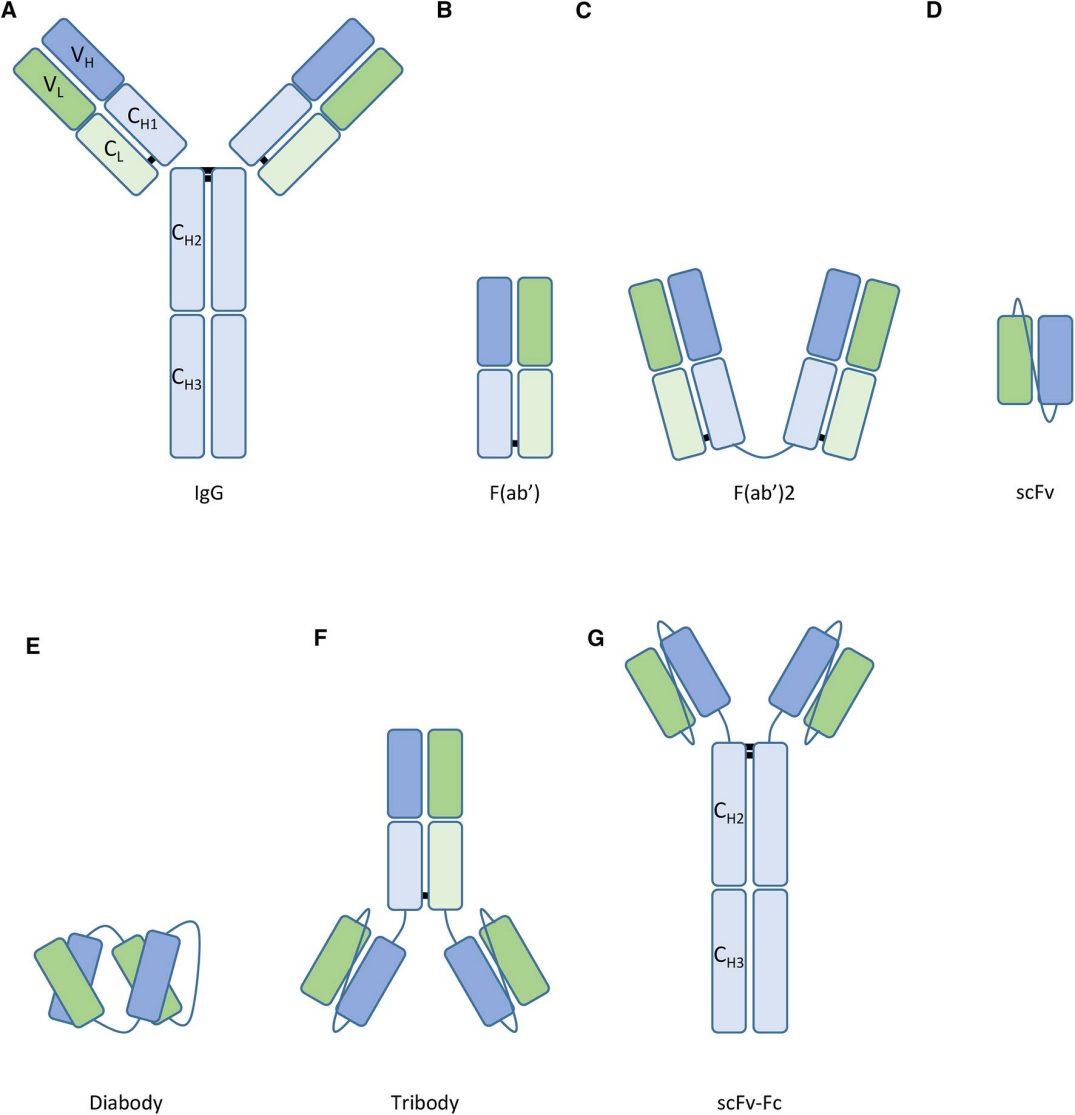
Schematic representation of antibody formats used in immunocytokines. Variable domain light chain (VL), constant region light chain (CL), variable domain heavy chain (VH), constant region heavy chain (CH), immunoglobulin G (IgG), antigen-binding fragment (F(ab′)), single chain variable fragment (scFv), fragment crystallizable region (Fc)

Immunocytokine formats can generally be divided into two categories: i.e. intact IgG or based on antibody fragments. Using intact IgG as delivery platform has several advantages (Table 1). Antibodies contain two light- and two heavy chains (Fig. 1A). Both light- and heavy chains have a variable domain (VL and VH), which together allow specific antigen binding. The light chain contains a single constant region (CL), while the heavy chain comprises three constant regions (CH1, CH2 and CH3). The first advantage of this format is the half-life of an IgG antibody, which is around 21 days in serum. This relatively long half-life is mediated by its large size (150 kDa), which reduces renal clearance rates [42, 43]. Additionally, antibodies of the IgG isotype are able to bind to the neonatal Fc receptor (FcRn), which prevents degradation of IgG in endothelial lysosomes and recycles them back into circulation [44]. Second, the dual variable regions of intact IgG grants immunocytokines based on this format high avidity for its specific target, which allows for high rates of retention at the site of interest [45]. High retention rates combined with long half-life provide IgG antibodies with a large window of activity. Third, IgG antibodies bind to Fc gamma receptors (FcγR’s), which are among others expressed on monocytes, macrophages and NK cells. This can lead to antibody-dependent cellular cytotoxicity (ADCC) or antibody-dependent cellular phagocytosis (ADCP) of the tumor cell, depending on the effector cell. Internalization of the immune complex also leads to degradation of the antibodies, reducing half-life. Monocytes and macrophages mostly kill via phagocytosis, while NK cells secrete perforins and granzymes, resulting in apoptosis [46, 47].

**Table 1:** The strengths and weaknesses of various antibody formats used in immunocytokines

	Size (kDa)	Strengths	Weaknesses
Intact IgG	150	Long serum half-life High target avidity Fc-mediated functions	Low tumor penetration Off-target toxicity
F(ab′)	48	High tumor penetration Decreased off-target toxicity	Low serum half-life Decreased avidity
F(ab′)2	96	Increased tumor penetration Decreased off-target toxicity	Low serum half-life
scFv	27	High tumor penetration Decreased off-target toxicity	Low serum half-life Decreased avidity
Diabody	54	High tumor penetration High target avidity Decreased off-target toxicity	Low serum half-life
Tribody	71	Increased tumor penetration High target avidity Decreased off-target toxicity	Low serum half-life
scFv-Fc	106	Long serum half-life High target avidity Fc-mediated functions	Decreased tumor penetration Off-target toxicity

However, not all FcγR’s are activating receptors. FcγRIIb, for example, contains an immunoreceptor tyrosine-based inhibitory motif (ITIM) and is commonly seen as an immune regulator for IgG-mediated activation of various immune cells [48]. In addition, FcγRIIb can also be expressed in a soluble form or cleaved from its transmembrane domain, allowing it to regulate immune responses through binding of immune complexes [49, 50].

Furthermore, high density opsonization of a tumor cell with IgG will attract C1q proteins, starting the complement cascade, which can lead to cell lysis via the membrane attack complex or phagocytosis by effector cells [51–53]. However, using an intact IgG antibody format also has some limitations. Its large size may hamper extravasation from vasculature and penetration into the tumor mass, preventing efficient localization at the site of cancer [54]. Additionally, if normal cells have (low) expression of the target antigen, binding of intact IgG may lead to ADCC or ADCP in healthy tissues. This, combined with the long half-life and high tissue retention may lead to higher toxicity in patients [55].

### Fragment-based formats

To improve tumor penetration, other antibody-based formats have been developed, such as F(ab’) fragments (Fig. 1B). Composed of the variable domains attached to the CH1 and CL subregions of the heavy and light chains, F(ab′) is essentially one truncated arm of an intact IgG antibody. The advantage of F(ab′) fragments is their smaller size (48 kDa) compared to the intact IgG format (150 kDa), which allows for increased extravasation and tumor penetration [56]. However, because the F(ab′) format only has a single antigen-binding domain, the avidity for its target is decreased. This disadvantage is not present in F(ab′)2 fragments, which are a variant in which two F(ab′) fragments are connected through a linker, restoring high avidity and retention at the site of interest (Fig. 1C). Furthermore, these fragments do not contain an Fc region, preventing binding of activating and inhibitory FcɣR’s, as well as FcRn, decreasing their half-life significantly. Decreased half-life in addition to the lack of Fc-mediated functions strongly reduce the off-target toxicity of these fragments.

The smallest fragment-based format is a single-chain variable fragment (scFv), consisting of the two variable domains of the heavy and light chain connected together with a short linker (Fig. 1D). scFv-based fragments have a molecular weight of approximately 27 kDa and are about six times smaller compared to intact IgG. This allows easier extravasation from the vasculature and higher penetration into the tumor mass. However, the small size and lack of Fc tail results in rapid renal clearance and a short half-life [42, 57, 58]. Like F(ab′) fragments, scFv’s only have a single antigen binding site, leading to decreased avidity to its target and a shorter retention time at the site of interest [42, 59, 60]. As such, this format is better suited for payload delivery than to induce tumor killing. Similar to the F(ab′) fragments, a short half-life in addition to the lack of any Fc-mediated functions strongly reduce off-target toxicity.

To overcome the decreased avidity of scFv’s, Dia- and Tribodies have been developed (Fig. 1E, F) [61, 62]. A Diabody is a dimeric variant of the scFv, while the Tribody combines the Diabody format with an additional F(ab′) fragment. With more antigen-binding moieties, Dia- and Tribodies each have increased avidity while still possessing increased tumor penetration capacity compared to intact IgG formats. The scFv-Fc format was developed to increase the capabilities of scFv to induce ADCC and ADCP (Fig. 1G). scFv-Fc molecules are composed of two scFv fragments that are directly attached to the CH2 and CH3 subregions of IgG heavy chains. As such, it is in essence an intact IgG format, which misses the constant regions of the fab arms, CL and CH1. This grants increased avidity to its target compared to scFv, restores Fc-mediated tumor-killing capabilities and increases half-life [63]. Nonetheless, restoration of these functions comes at the cost of decreased tumor penetration due to a larger size (106 kDa) and increased off-target toxicity if the target is expressed in healthy tissue.

## Immunocytokines in (pre-)clinical trials

There is a good number of interesting immunocytokines currently in clinical development (Table 2). In the next paragraphs, we will briefly discuss the key players currently in (pre-) clinical development.

**Table 2:** List of immunocytokines currently in clinical development

Schematic	Name	Antigen		Antibody format	Payload	Combined with	Patients	Status	Phase	ID
	FAP-IL2v (RO6874281)	FAP		Intact IgG	IL-2v	Trastuzumab/cetuximab	Solid tumors	Active	Ia/Ib	NCT02627274
	FAP-IL2v (RO6874281)	FAP		Intact IgG	IL-2v	Pembrolizumab	Metastatic Melanoma	Active	Ib	NCT03875079
	FAP-IL2v (RO6874281)	FAP		Intact IgG	IL-2v	Various immunotherapy-based pre-treatments	Pancreatic Adenocarcinoma	Recruiting	Ib/II	NCT03193190
	F16-IL2 (Teleukin)	Extra-domain A1 of tenascin-C		scFv	IL2	Cytarabine	Relapsed acute myeloid leukemia	Active	I	NCT02957032
	Hu14.18-IL2	GD2		Intact IgG	IL-2	Ex vivo expanded donor NK cells	Neuroblastoma/Osteosarcoma	Suspended	I	NCT03209869
	Hu14.18-IL2	GD2		Intact IgG	IL-2	Nivolumab/Ipilimumab/radiation therapy	Melanoma	Suspended	I/II	NCT03958383
	L19-IL2 (Darleukin)	EDB		scFv	IL-2	DTIC	Metastatic melanoma stage IV	Active	I/II	NCT02076646
	L19-IL2 (Darleukin)	EDB		scFv	IL-2	Rituximab	Diffuse large B cell lymphoma	Active	I/II	NCT02957019
	L19-IL2 (Darleukin)	EDB		scFv	IL-2	Radiation therapy	NSCLC stage IV/metastatic disease	Recruiting	II	NCT03705403
	L19-IL2 + L19-TNF (Daromun)		EDB	scFv	IL-2 + TNF-alpha (monomer)	–	Basal cell carcinoma/cutaneous squamous cell carcinoma	Recruiting	II	NCT04362722
	L19-IL2 + L19-TNF (Daromun)		EDB	scFv	IL-2 + TNF-alpha (monomer)	Surgery	Stage III B/C melanoma	Recruiting	III	NCT02938299
	L19-IL2 + L19-TNF (Daromun)		EDB	scFv	IL-2 + TNF-alpha (monomer)	Surgery/adjuvant therapy	Stage III B/C melanoma	Recruiting	III	NCT03567889
	L19-TNF (Fibromun)		EDB	scFv	TNF-alpha (monomer)	–	Grade III/IV glioma	Active	I/II	NCT03779230
	L19-TNF (Fibromun)		EDB	scFv	TNF-alpha (monomer)	Lomustine	Glioblastoma	Recruiting	I/II	NCT04573192
	L19-TNF (Fibromun)		EDB	scFv	TNF-alpha (monomer)	Temozolomide	Glioblastoma	Recruiting	I/II	NCT04443010
	L19-TNF (Fibromun)		EDB	scFv	TNF-alpha (monomer)	Dacarbazine	Soft Tissue Sarcoma	Recruiting	II	NCT04733183
	L19-TNF (Fibromun)		EDB	scFv	TNF-alpha (monomer)	Doxorubicin	Leiomyosarcoma	Recruiting	II	NCT03420014
	L19-TNF (Fibromun)		EDB	scFv	TNF-alpha (monomer)	Doxorubicin	Soft Tissue Sarcoma	Recruiting	III	NCT04650984
	NHS-IL12 (M9241)		DNA/histone complex	Intact IgG	IL-12	Bintrafusp Alfa/radiation therapy	Stage IV breast cancer/breast (adeno) carcinoma	Recruiting	I	NCT04756505
	NHS-IL12 (M9241)		DNA/histone complex	Intact IgG	IL-12	Bintrafusp Alfa / radiation therapy	Metastatic non-prostate genitourinary cancers	Recruiting	I	NCT04235777
	NHS-IL12 (M9241)		DNA/histone complex	Intact IgG	IL-12	ADT/prednisone/bintrafusp alfa/docetaxel	Prostate cancer	Recruiting	I/II	NCT04633252
	NHS-IL12 (M9241)		DNA/histone complex	Intact IgG	IL-12	Bintrafusp Alfa	Kaposi Sarcoma	Recruiting	I/II	NCT04303117
	NHS-IL12 (M9241)		DNA/histone complex	Intact IgG	IL-12	Bintrafusp alfa/CV301/N-803	Colorectal cancers/small bowel cancers	Recruiting	II	NCT04491955
	L19-IL12 (Dodekin)		EDB	scFv	IL-12	-	Advanced or metastatic carcinoma/diffuse large B cell lymphoma	Recruiting	I	NCT04471987

IL-2 is a key cytokine for the differentiation and activation of cytotoxic effector cells, including CTLs, NK cells and macrophages. However, systemic treatment with IL-2 results in severe toxicity [36].

Immunocytokines with an IL-2 moiety increased leukocyte infiltration within the TME of murine tumors. Strong NK cell induced antitumor immunity was observed, leading to necrosis and decreased tumor cell proliferation without the high rates of toxicity associated with untargeted IL-2 therapy [64–67].

**Fig. 2:**
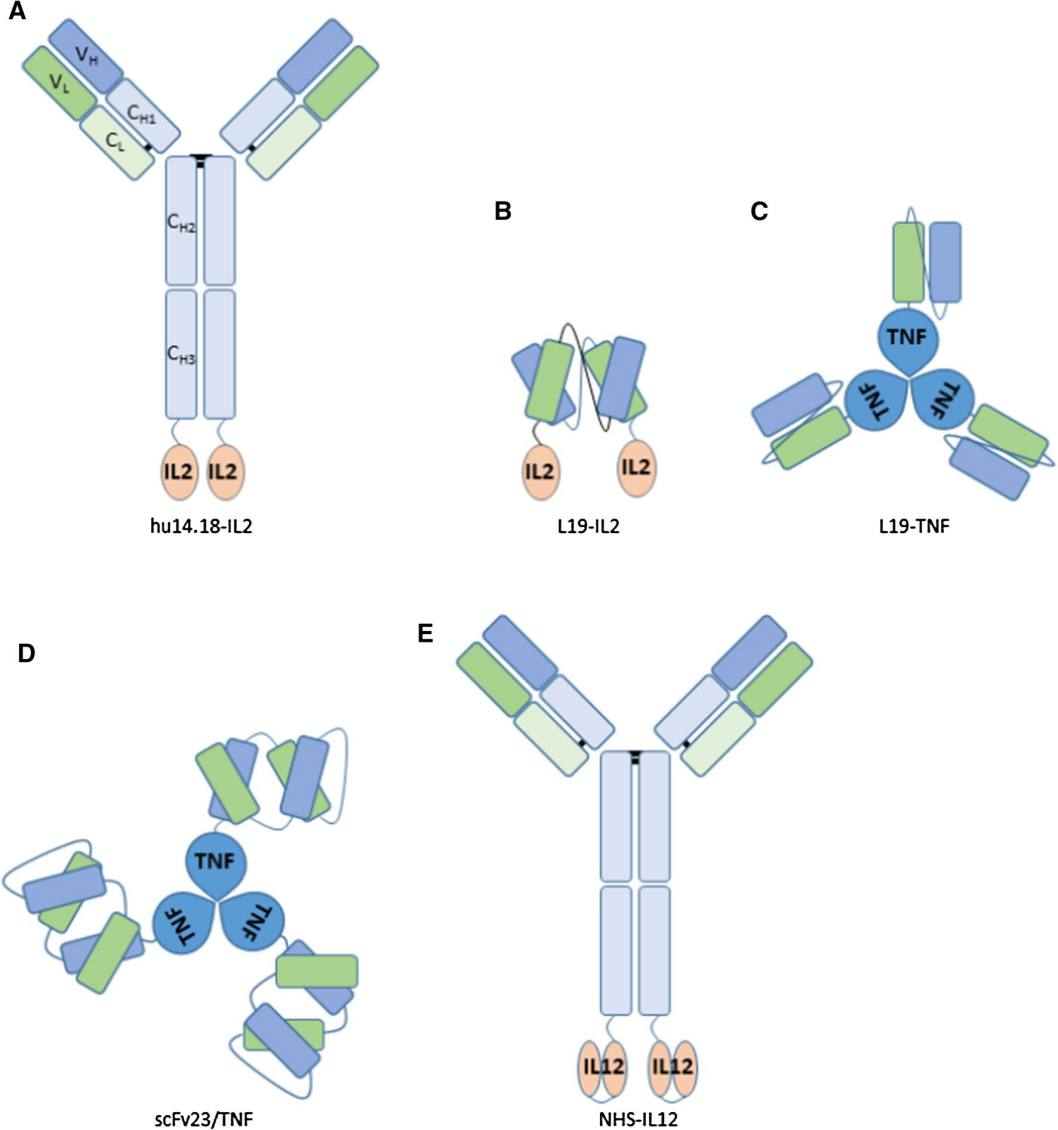
Schematic representation of immunocytokine formats in (pre-)clinical trials

Several IL-2 immunocytokines have therefore been tested in clinical studies, with promising results. One of these immunocytokines is hu14.18-IL-2. This immunocytokine is based on the hu14.18 antibody clone, which is a humanized antibody against GD2, a tumor antigen which is overexpressed in neuroblastoma as well as melanoma and highly restricted on normal tissues with the exception of the immune-privileged cerebellum and peripheral nerves. It has an intact IgG format, with IL-2 molecules genetically linked at the C-terminal of both heavy chains (Fig. 2A). Fusing two IL-2 molecules has the advantage of increased immune modulation compared to immunocytokines with only one IL-2 molecule. The intended method of action for this immunocytokine is to locally activate NK cells and CTLs through the IL-2 payload, and induce ADCC via the functional IgG Fc region. The activation of CTLs makes this immunocytokine an excellent candidate for combination with other therapies aimed at inducing adaptive immune responses, such as vaccination or immune checkpoint inhibitors [68, 69]. In a phase I/II clinical trial, increased numbers of NK cells were found in circulation [70]. Isolated patient NK cells (before treatment), were more effective in inducing ADCC in the presence of hu14.18-IL-2. Moreover, significantly less side effects of hu14.18-IL-2 treatment were observed when compared with untargeted IL-2 therapy, which was confirmed in another clinical study [71]. Consequently, significantly higher dosage can be given, which likely improves efficacy of IL-2 treatment [72]. Intravenous treatment with hu14.18-IL-2 after complete resection of stage III or IV melanoma resulted in prolonged tumor-free survival of patients with high risk of recurrence [73].

Additionally, a variant (IC35) was developed due to dose-limiting toxicity that was experienced when the hu14.18-IL2 immunocytokine was used clinically. In this variant, the payload was genetically fused to the light chains instead of the C-terminal of the heavy chains using the same linker as the original [74]. It was anticipated that this would result in decreased interaction of IL-2 payloads with their receptors due to increased steric hindrance of the antibody, which, in turn, would decrease off-target toxicity. Pre-clinical data showed that IC35 had decreased affinity for βɣIL2R as well as reduced ability to induce IL-2-mediated proliferation in multiple cell lines, supporting the hypothesis.

L19-IL-2, which targets extradomain-B (EDB; i.e. alternatively spliced fibronectin) represents another widely tested IL-2 immunocytokine. EDB is primarily expressed in embryos and on tumor vasculature [75, 76]. Choosing a TAA that is not expressed on the tumor cells itself, but on the tumor vasculature changes the therapy dynamics, allowing for a different approach for tumor elimination. L19-IL-2 has a non-covalent homodimeric scFv format, allowing for more efficient penetration into the TME as well as a genetic fusion of two IL-2 molecules via (SSSSG)_3_ linkers (Fig. 2B). Similar to hu14.18-IL-2, L19-IL-2 can locally activate NK cells and CTLs via the IL-2 payload, but lacks the capacity to induce ADCC due to the missing Fc region. Nonetheless, without a functional Fc region, off-target toxicity of the immunocytokine is also reduced. NK cells were the main effector cells for induction of tumor cell killing in a pre-clinical pancreatic tumor model [64]. Furthermore, L19-IL-2 monotherapy induced tumor growth delay of 8 days in a Ramos lymphoma xenograft model, whereas combination with Rituximab (anti-CD20 mAb) therapy resulted in completely tumor eradication [77]. No irreversible toxicity was developed when patients with metastatic renal carcinoma were treated with L19-IL-2 in a Phase I/II trial [78]. L19-IL-2 treatment, in combination with chemotherapy (dacarbazine) lead to an increased number of partial responses and decreased progressive disease in patients with metastatic melanoma [79, 80].

L19-IL-2 has also been extensively tested in combination with L19-TNF, an immunocytokine that is composed of the same scFv targeting EBD, but genetically linked with a different cytokine, i.e. TNF, using (SSSSG)_3_ linkers (Fig. 2C). L19 scFv does not homodimerize, but trimerizes due to the trimerization motifs present in the TNF payload. As a result, a functional, trimerized immunocytokine has three scFv moieties and one active TNF homotrimer. This increases binding avidity and decreases off-target toxicity through increased retention at the tumor site. Combining L19-IL-2 and L19-TNF treatments resulted in complete remission in murine F9 carcinoma models, while the individual monotherapies were unable to completely eradicate tumors [81]. Similar results were found in a murine myeloma model, in which the combination treatment eradicated 58% of tumors [82]. The combination of L19-IL-2 and L19-TNF was also tested in a phase II clinical trial, in which this treatment showed remarkable efficacy in eradicating non-injected melanoma lesions (7/13 lesions) [83].

Other TNF-based immunocytokines have been developed, but have not reached clinical stage yet. Nevertheless, pre-clinical results of the scFv23/TNF immunocytokine were encouraging. The scFv23 fragment-based immunocytokine, with a Diabody format, targets HER-2/neu, which is overexpressed in approximately 30% of breast cancers (Fig. 2D). The scFv23/TNF is genetically fused to a single TNF monomer using a GGGGS linker, similar to the L19-TNF immunocytokine. As such, it also trimerizes at the target site. The putative mode of action is sensitization of HER-2/neu overexpressing breast cancer cells to TNF-therapy and subsequent induction of apoptosis through the direct effects of TNF on tumor cells. HER-2/neu overexpressing breast cancer cells are typically resistant to the cytotoxic effects of TNF. However, treatment with scFv23/TNF increased expression of the TNFR1 by 5- to sevenfold and effectively sensitized the cells to TNF-induced apoptosis [84]. This principle was confirmed when scFv23/TNF was tested on HER-2/neu overexpressing pancreatic cancer cell lines in combination with various chemotherapeutics. Cell lines that were originally resistant to TNF-induced apoptosis, were killed in equal measure by scFv23/TNF monotherapy when compared to conventional chemotherapeutic agents like 5-fluorouracil or etoposide [85].

Additionally, IL-12-based immunocytokines have also been evaluated in clinical studies. The NHS-IL-12 immunocytokine is based on intact IgG and has two IL-12 heterodimers genetically fused to its heavy chain C-terminals via a flexible (GGGGS)_3_ linker. The variable domains target DNA/histone complexes, which are not present extracellularly during homeostasis (Fig. 2E). By contrast, DNA/histone complexes are often found at sites of necrosis such as a necrotic tumor core. When tumor necrosis was induced via local irradiation in a human rhabdomyosarcoma xenograft mouse model, increased localization of NHS-IL-12 was observed at the site of the tumor [86]. Since IL-12 plays an important role in crosstalk between the innate and the adaptive immune system, the intended mechanism of action for this immunocytokine is to stimulate antigen-presenting cells, CD8+ T cells and NK cells, thereby initiating robust antitumor adaptive immune responses. When MC38 tumor-bearing mice were treated with various doses of NHS-IL-12, increased serum IFN-ɣ levels were found as well as splenic DC maturation [87]. Additionally, treatment of subcutaneous LLC, B16 and MC38 tumors in athymic mice led to significant growth reduction in all cases. A phase I clinical trial showed that all dose-limiting responses were transient and levels of circulating activated NK and NKT cells were increased after treatment [88]. A phase I/II clinical study investigating the combination treatment with M7824, an anti-PD-L1 antibody carrying a TGF-β trap (the extracellular domain of human TGFβRII), has been initiated (NCT04303117).

## Current challenges and routes of future antibody development

Improvement of immunocytokines can be achieved in two ways, i.e. innovation of the antibody moiety or of the payload. Adaptation of the antibody might represent the easier route, since many advancements or concepts in the field of antibody engineering have already been made. Any attempts to improve the efficacy or to decrease side-effects of mAb therapy can practically be directly translated into the field of immunocytokines.

### Point mutations

Simple amino acid mutations were shown to have significant effects on the efficacy and/or pharmacokinetics of antibodies [89]. However, they have not been applied in the field of immunocytokines yet. On the one hand, point mutations can be used to increase the effector function of immunocytokines, such as immune cell and complement activation via the Fc tail. FcyRIII is involved in the initiation of ADCP or ADCC by monocytes and macrophages or NK cells. Via extensive mapping of the binding site of IgG Fc receptors, amino acid mutations were identified that increased binding of FcyRIIIA, leading to a twofold increase in ADCC [90]. The triple mutation Ser293Asp/Ala330Leu/Ile332Glu increased both ADCP and ADCC [91]. The same mutation also enhanced the efficiency of NK cell-mediated serial killing [92]. The initial step of the classical complement cascade is binding of C1q to the CH2 region of IgG [93]. Residue mutations that increase the binding of this first step may therefore prove beneficial to the efficacy of the antibody and immunocytokines. As examples, Lys326Ala/Glu333Ala or Lys326Met/Glu333Ser mutations increased complement-dependent-cytotoxicity (CDC) without interfering with the antibody’s ability to induce ADCC [94]. Another application of residue mutations is optimization of the FcRn-binding region. One such mutation increased the half-life by 3,2- or 3,onefold of bevacizumab (IgG1) or cetuximab (IgG1) respectively [95]. A longer half-life increases the therapeutic potential of therapies and reduces the cost of treatment. However, making amino acid mutations in the FcRn-binding region of antibodies might have adverse effects on its capabilities to mediate ADCC, which also has to be addressed [96].

On the other hand, when Fc tail-initiated functions in fact cause harm, amino acid mutations can also be used to reduce off-target toxicity. Over-activation of immune cells or activation of immune cells in healthy tissues can result in significant damage, precluding clinical development. Additional off-target effects can include depletion of immune cells. Either tumor-infiltrating immune cells are purposefully targeted by bispecific antibodies for activation or immune cells express the same target receptors present on tumor cells (e.g. CD20 on B cells). In either case, opsonization of immune cells can mark them for Fc-mediated killing via NK cells or macrophages, resulting in depletion and immune-compromised patients. A potential solution is to use an antibody format that lacks the Fc region, such as scFv and F(ab′) fragments, decreasing the half-life as well as retention at the site of disease, which may diminish side effects. Silencing of Fc regions by residue mutations represents an alternative approach, which retains the long half-life of FcRn-binding antibodies, but abolishes the possible off-target toxicity mediated by activation of immune cells or complement via the Fc region. The most commonly used mutations in this approach are the Leu234Ala and Leu235Ala (LALA) mutations, which for instance abrogated binding of the IgG1 isotype OKT3 antibodies to FcyRI, FcyRIIa, and FcyRIIIa [97–99].

### Glyco-engineering

It is also possible to change the pharmacokinetics of immunocytokines using glyco-engineering, which is regulating post-translational glycosylation of proteins as opposed to changing the genetic code in the case of residue mutations. Increased formation of a bisecting GlcNAc structure in *N*-glycans, through the expression of b(1,4)-*N*-acetylglucosaminyltransferase-III in IgG1-producing cells, increased FcyRIII-mediated ADCC by 10- to 20-fold due to increased affinity of the antibody’s Fc region [100, 101]. Increased galactosylation of antibodies enhanced the capabilities of IgG1 antibodies to induce CDC [102]. The same effect was observed for IgG3 antibodies, while increased galactosylation did not affect the ability to induce CDC of IgG2 and IgG4 isotypes [103]. Several amino acid mutations also had effect on the glycosylation profile, which proved effective in the silencing of ADCC and/or CDC capabilities of antibodies. Substitution of asparagine at position 297 by either alanine, glutamine, or glycine removed this glycosylation site and dramatically decreased the ADCC and CDC capabilities of IgG1 and IgG3 antibodies [104–107].

### Bi-specific formats

**Fig. 3:**
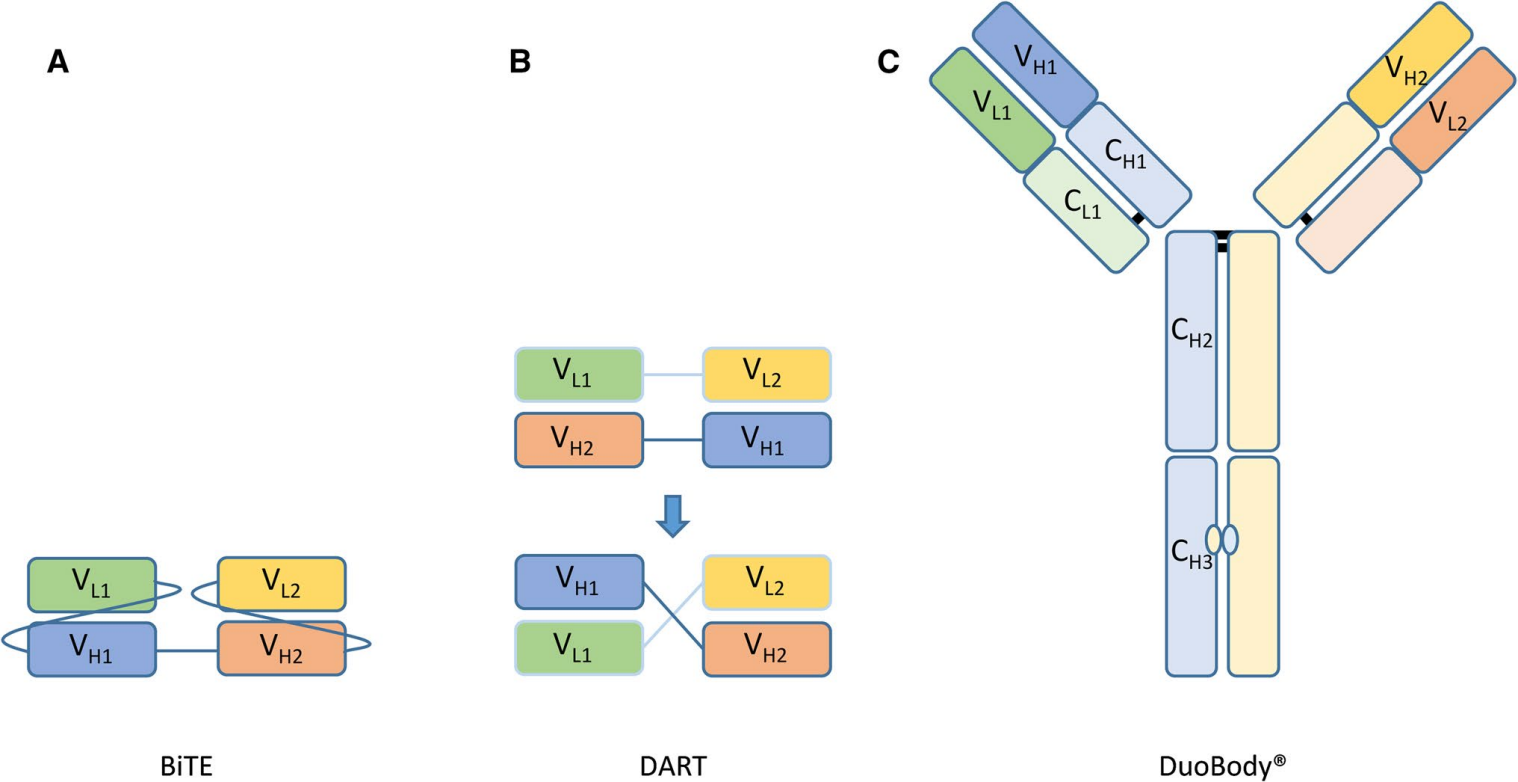
Schematic representation of bispecific antibody formats. Abbreviations used: variable domain light chain (VL), constant region light chain (CL), variable domain heavy chain (VH), constant region heavy chain (CH). Bispecific T cell Engagers (BiTEs), Dual-Affinity Re-targeting proteins (DARTs)

Bi-specific antibodies (BsAbs) can be used to recruit effector cells populations, which are normally not effectively engaged by IgG-based antibody therapy. For instance, CTLs (CD8 T cells) are not involved in antibody therapy as they lack Fc receptors. As CD8 cells have great capacity to kill tumor cells, they could significantly add to antibody-based therapy efficacy. T cell activating BsAbs generally target the CD3 receptor in combination with a tumor-associated antigen (TAA) [108–110]. Bispecific T cell Engagers (BiTEs), Dual-Affinity Re-Targeting proteins (DARTs) and DuoBody® formats have been described. The BiTE format simply consists of two scFv’s that target different antigens, linked together as a single chain (Fig. 3A). The DART format was designed to overcome some of the limitations of BiTEs. It consists of two separate proteins, in which one contains the VH against antigen 1 and the VL of antigen 2, while the other contains the VH against antigen 2 and the VL of antigen 1 (Fig. 3B). This format allows the heterodimerization that naturally occurs in IgG to form a functional bispecific antibody. The DuoBody® format is an intact IgG format that consists of a complete IgG molecule with two different antigen binding domains (Fig. 3C). This is achieved by generating two separate parental antibodies and performing a Fab-arm exchange using DuoBody® technology [111].

Alternatively, neutrophils are the most abundant cytotoxic effector cells in the circulation. It was demonstrated by us and others that engaging the IgA Fc receptor (FcαRI) on neutrophils effectively induced neutrophil recruitment and tumor cell killing [112–114]. However, IgA has shorter half-life (± 6–7 days) compared to IgG. Recently a DuoBody® IgG1 BsAb was developed of which one of the variable domains binds a tumor antigen, while the other variable domain binds FcαRI [115]. This BsAb was able to recruit neutrophils (via the FcαRI arm) and NK cells and macrophages (via the IgG1 Fc tail) as effector cells in a B16F10 melanoma mouse model [115]. Similar effects can be achieved by genetically fusing IgG and IgA Fc regions or by creating a chimeric variant of the two regions (IgGA) [116–118].

Bi-specific formats can also be used to further increase activation of immune cells sensitive to FcɣR stimulation. NK cells, for example, can bind IgG, but are further activated by agonistic antibodies that activate a co-receptor [119]. Primary targets for agonistic activation by antibodies include NKG2D, 2B4 and NKp30 [120–122]. BsAb immunocytokines may be used to further activate effector cells of interest. However, bispecific formats can also be used to increase specificity for the tumor by targeting two tumor antigens instead of one. Several tumor antigens that are used for mAb therapy are overexpressed in tumors, but also expressed in healthy tissues, leading to off-target toxicity. Targeting immunocytokines toward two antigens that are overexpressed in tumor tissue may reduce off-target toxicity [123].

### Tumor-associated antigen expression in patients

The translation of target-specificity from pre-clinical to clinical models is a serious challenge. Commonly, pre-clinical models are used that either strongly overexpress a (TAA) or express a human TAA that is not found in the used animals. In the first instance, the expression of TAA might be artificially inflated compared to clinical tumors, and care must be taken to ensure that the therapy is still sufficiently specific to prevent off-target toxicity when the TAA expression is lower. Similarly, in the case of expressing a human TAA in an animal model, unwanted off-target toxicity may be missed as the target is not expressed in normal tissues. Additionally, using a human TAA in an animal model will increase the immunogenicity of the tumor, possibly confounding the therapeutic effect in patients.

## Current challenges and routes of future payload development

### Alternative payloads

Most cytokine payloads that are currently tested in a clinical setting are pro-inflammatory, aiming to activate or stimulate immune cells [45]. Alternatively, anti-inflammatory cytokines may be considered. Previously, a Cetuximab-based immunocytokine with IL-10 as payload (CmAb(IL-10)_2_) was developed, which is classically considered as an anti-inflammatory cytokine [124]. Interestingly, treatment with CmAb(IL-10)_2_ in several murine tumor models, resulted in superior tumor growth reduction compared to treatment with Cetuximab alone. CmAb(IL-10)_2_ mediated its effects by hindering IFN-ɣ-induced cell death of tumor-infiltrating CD8+ T cells. This effect was even more pronounced when CmAb(IL-10)_2_ treatment was combined with checkpoint inhibitors such as anti- PD-L1 and anti-CTLA-4 mAbs.

Although counterintuitive, these data demonstrate that anti-inflammatory cytokines can also (indirectly) induce pro-inflammatory effects. This concept was previously proposed in a review in which it was described that the inflammatory effect of cytokines depends on multiple factors, such as dosage, target cells and experimental models. As such, cytokines cannot be defined in a black-and-white manner like pro- or anti-inflammatory, and it might therefore be worthwhile to further investigate immunocytokines with classically anti-inflammatory cytokines as payload [125].

Another cytokine subset worth looking into is the chemokine family. Chemokines form a complex signaling network with their receptors that allow for the chemotaxis of specific cell subsets toward a gradient of chemokines [126]. In spite of a lot of promiscuity in the chemokine network, there are some chemokines that specifically (or rather preferentially) attract cell subsets. Using immunochemokines to create a local chemokine gradient in the tumor can therefore attract specific immune cells of interest. This may allow for specific skewing of immune responses, which could be of great value for immunotherapy. Recent findings have shown that the tumor manipulates the chemokine milieu to its own advantage by attracting immune cells that are converted into tumor-associated regulatory immune cells [127]. Using chemokines as payload might provide an opportunity to rebalance the chemokine milieu and the immune cell infiltrate.

However, several aspects need to be taken into account when developing immunochemokines. First, the N-terminus of chemokines plays a key role in the interaction with their respective receptors [128, 129]. Fusing chemokines to an antibody might interfere with that interaction. Similarly, chemokines are usually presented on glycosaminoglycans (GAGs) to circulating immune cells by endothelial cells [130, 131]. Having a chemokine presented via an antibody instead of GAGs may potentially severely impair the ability of chemokine receptors to recognize and bind the chemokine moiety of an immunochemokine. Both of these problems might be solved by attaching the chemokine via a cleavable linker. After cleavage, the chemokine is released from the antibody moiety. This allows the chemokine to be presented on GAGs, while also freeing up its N-terminus for interaction with chemokine receptors. Cleavable linkers can be cleaved by matrix metalloproteinases (MMPs) secreted specifically by tumor cells [132], which prevents systemic release of the chemokine. After cleavage, the antibody moiety of the immunochemokine is still able to bind target receptors and mediate tumor killing through its Fc tail.

### Potency matched dual-cytokines

A handful of immunocytokines have already proven their efficacy in clinical trials when used as a mono-therapy [73, 79, 80, 88]. However, some immunocytokines, which carry multiple different payloads have been developed. These dual-cytokine immunocytokines have currently only been tested in in vitro and pre-clinical models [133–138]. With the cytokine repertoire’s scale and variety, many combinations can be made which could have synergetic effects in vivo. Nonetheless, currently cytokine combinations have been limited to IL-2/TNF, IL-2/TNFR2 and IL-2/TRAIL, which all show remarkable promise.

When designing dual-cytokine immunocytokines, it is important to realize that most cytokines have a different effective dose. When both cytokines are fused to an antibody, the amount of cytokines will be averaged, which may result in an ineffective dose of one of the cytokines. Alternatively, too much of one cytokine may lead to toxicity. Changing the affinity of one of the cytokines for their receptor to match the other cytokine may overcome this issue [135].

### Reduction of payload-mediated toxicity

Generally, off-target toxicity is mediated through the Fc region of the antibody moiety. However, toxicity might also be mediated through the payload of an immunocytokine. To overcome this challenge, multiple methods can be used.

**Fig. 4:**
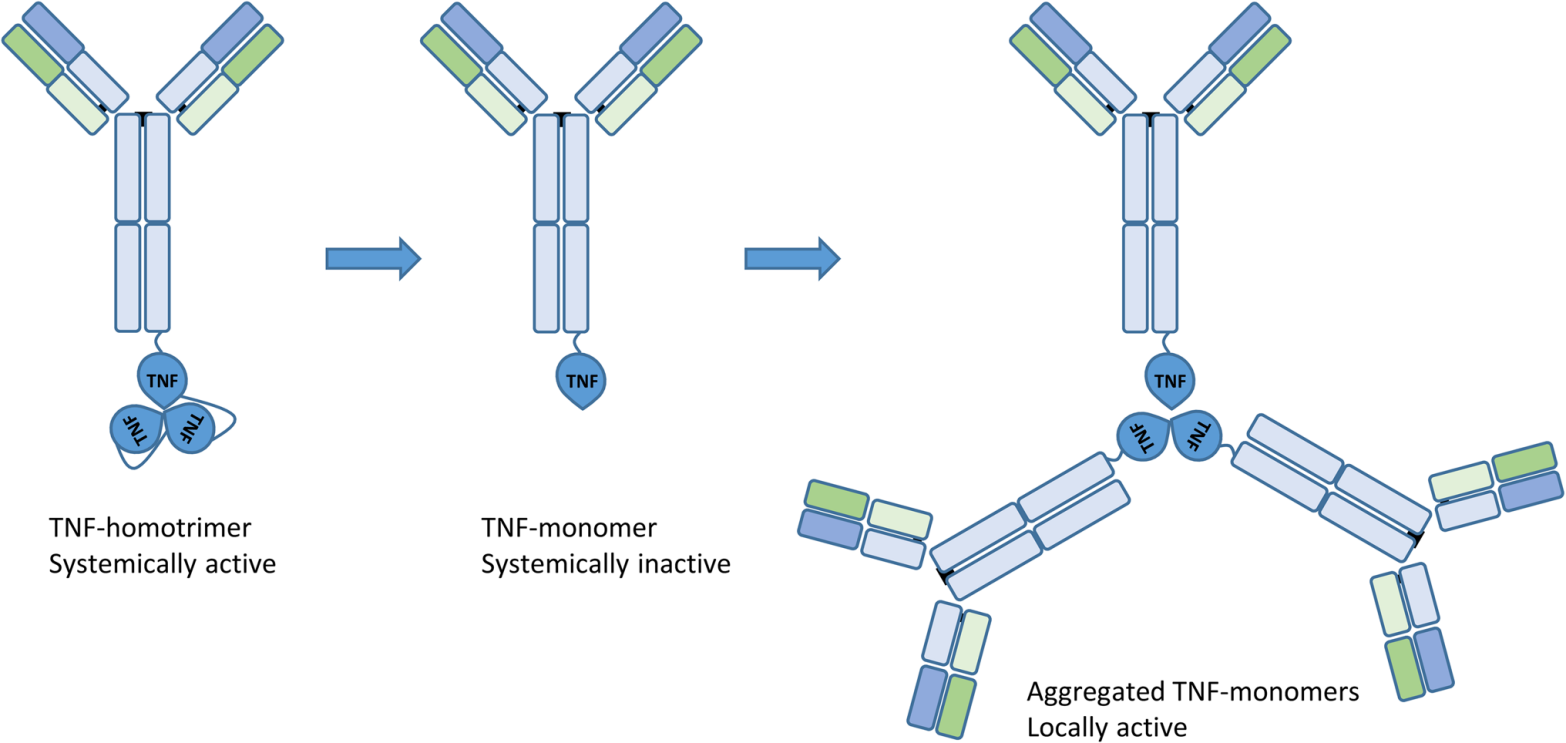
Schematic overview of the prevention of payload-mediated toxicity via the separation of an active TNF-homotrimer into inactive TNF-monomers. This allows the formation of the active homotrimer at sites where the antibodies aggregate, i.e. the tumor micromilieu

One method to reduce payload-mediated toxicity can be applied if the active form of the cytokine in question is a multimer. In the case of the L19-TNF immunocytokine for example, TNF-α is only active in its homo-trimeric form [139]. Developing an immunocytokine with a TNF-α homo-trimer as payload is possible, but might induce toxicity when injected systemically. Developing an immunocytokine with only a monomer as payload may represent a solution, as the active TNF-α homo-trimer is only formed when the immunocytokine localizes to the tumor (Fig. 4) [81]. A similar approach may be used for an IL-12 payload. IL-12 is a heterodimer composed of a p35 and p40 subunit. Constructing two immunocytokines with the p35 and p40 payloads respectively will ensure that the active form of IL-12 is only formed at the site of localization [140].

A relatively recent development in toxicity reduction is protease-cleavable epitope masking. The basic principle behind this technique is to link an epitope-masking molecule to the antibody via a linker that is cleavable by MMPs, which are specifically or highly expressed at tumor sites [141]. Using this technique, immune-active antibody moieties, such as Fc regions, binding domains, receptor agonistic variable domains or payloads, can be kept in an inactive state until the antibody reaches the tumor [142–144]. Once arrived, tumor-specific proteases cleave the linker, releasing the masking molecule from the antibody and converting it to an active state.

Similar to including point mutations to reduce antibody-mediated toxicity, this technique has also been successfully applied to cytokines [145, 146]. These mutated cytokines are referred to as Activation-by-Targeting Cytokines (AcTakines). By mutating amino acid residues involved in receptor-ligand interactions, the affinity of the cytokine for its receptor can be reduced, which leads to decreased signaling at lower concentrations, such as in the circulation or in non-targeted tissues. However, increased concentrations of the immuncytokine in tumors are sufficiently high to enhance avidity to a level which allows efficient signaling.

Alternatively, small molecule inhibitors have been used to temporarily inhibit the respective signaling pathway of the cytokine receptor [147]. This attenuates payload-mediated toxicity in the circulation. As the immunocytokine localizes at the tumor site, its payload becomes active once small molecules have been cleared from the body, reducing off-target toxicity.

## Conclusion

Immunocytokines provide a robust and versatile platform for the treatment of cancer. The flexibility of both antibody platforms and payload moieties ensures that immunocytokines can be developed to suit the specific characteristics of each type of cancer. Additionally, there is room for significant innovation and improvement of all aspects of immunocytokines, which may increase their efficacy even further. Thus, we anticipate exciting new developments in this field in the next few years.

## Data Availability

All relevant data can be found in the original references.
